# Furrowed tongue, fatten lip and facial droop

**DOI:** 10.11604/pamj.2020.36.325.25052

**Published:** 2020-08-24

**Authors:** Philip Babatunde Adebayo, Hanifa Mbithe

**Affiliations:** 1 Neurology Section, Department of Medicine, Aga Khan University, Dar es Salaam, Tanzania

**Keywords:** Facial palsy, furrowed tongue, Melkersson-Rosenthal syndrome

## Image in medicine

Melkersson-Rosenthal syndrome (MRS) is a rare, neuro-mucocutaneous syndrome characterized by recurrent facial nerve palsy, swelling of the upper lip, and fissured tongue. Young adults in the second and third decades are more predisposed. We report a case of a 23-years old female Chinese who presented with 5 days history of right facial weakness; her second event in one year (first weakness was on the left). On examination, she had right peripheral facial nerve palsy (House-Brackmann stage IV) and, mild swelling of the upper lip (Image-Arrow A). Her tongue revealed two central furrows. The anterior central furrow (Image-Arrow B) measured about 2cm and the posterior one measured about 1.5cm with lateral grooves and few posterior perpendicular furrows measuring about 1-2 mm in depth. Her complete blood count, blood sugar, urine routine, C-reactive protein, serum angiotensin-converting enzyme level was all normal. Her brain magnetic resonance imaging revealed no abnormal signals. She was commenced on oral prednisolone 60mg daily for 5 days, acyclovir 800mg 4 times daily for 5 days, vitamin B capsules and methylcellulose drops to prevent scleritis. She was commenced on physiotherapy with steady clinical improvement. Although fissured tongue is a common clinical condition with a long list of differential diagnosis, the presence of recurrent facia nerve palsy and swollen lip should raise the suspicion of MRS. Other causes of recurrent facial nerve palsy and buccal mucosa involvement like sarcoidosis, system lupus erythematosus, Bechet disease and Crohn disease should be considered.

**Figure 1:**
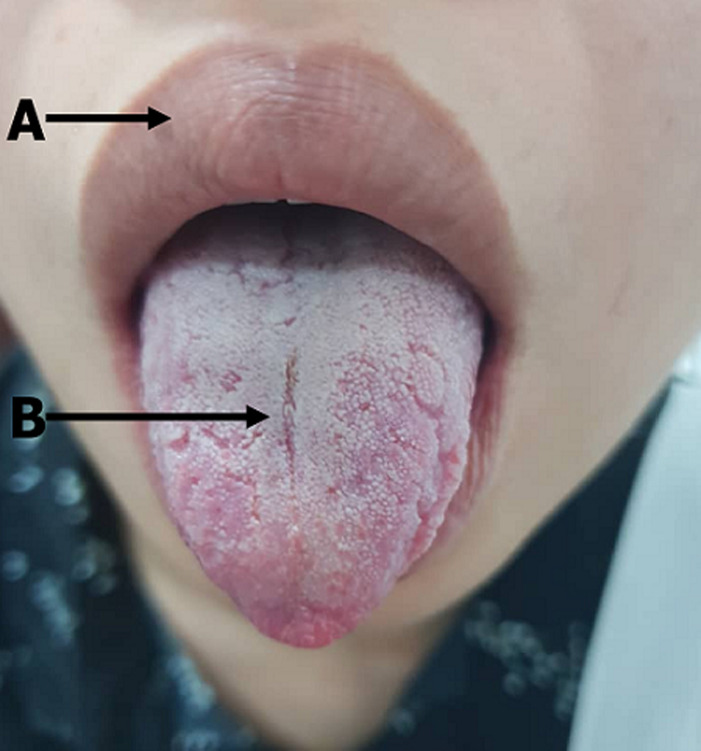
A) swelling of the upper lip; B) anterior central tongue furrow

